# Three sympatric host nestlings eavesdrop on cuckoo nestling distress calls

**DOI:** 10.1002/ece3.11437

**Published:** 2024-05-16

**Authors:** Jiaojiao Wang, Qindong Zhou, Taijun Zuo, Longwu Wang, Laikun Ma, Jianhua Hou

**Affiliations:** ^1^ College of Life Science, Hebei University Baoding China; ^2^ Engineering Research Center of Ecological Safety and Conservation in Beijing‐Tianjin‐Hebei (Xiong’an New Area) of MOE Baoding China; ^3^ School of Life Sciences Guizhou Normal University Guiyang China; ^4^ Department of Biology and Food Science Hebei Normal University for Nationalities Chengde China

**Keywords:** brood parasitism, common cuckoo, distress calls, playback, predation

## Abstract

In predator–prey interactions, the prey faces extreme challenges from predation, which drives the evolution of defense or anti‐predator mechanisms. Compared with adult birds, nestlings are more vulnerable but not helpless. However, data on whether nestlings eavesdrop on the danger signals transmitted by other prey nestlings and the mechanisms of eavesdropping remain limited. In brood parasitism, common cuckoo (*Cuculus canorus*) nestlings, raised by host adults who are not closely related, offer an instructive system for studying the transmission and recognition of danger signals among nestlings of different species that share special relationships. We played back the distress calls of common cuckoo nestlings to nestlings of three sympatric host species (the oriental reed warbler *Acrocephalus orientalis*, which is a primary host of the common cuckoo, the reed parrotbill *Paradoxornis heudei*, an occasional host, and the vinous‐throated parrotbill *Sinosuthora webbiana*, which is not parasitized in the study area) to investigate whether the host nestlings reduced their begging behavior. We also quantified the degree of inhibition toward begging behavior for these nestlings. The results revealed that, in response to the distress calls, the three sympatric host species markedly suppressed their begging behavior. This response can likely be attributed to the innate response of host nestlings caused by the general characteristics of distress calls, rather than the acoustic similarity and phylogenetic relationship between host nestlings and cuckoo nestlings. Furthermore, we observed that upon hearing the distress calls of cuckoo nestlings, the oriental reed warbler nestlings exhibited the greatest reduction in the total number of calls compared to the other two host species, potentially owing to stronger predation and parasitic pressures. This study suggests that host nestlings can detect danger signals emitted by parasitic nestlings; however, further investigation is needed to determine whether they can respond to distress calls from unfamiliar nestlings in different regions.

## Graphical abstract

Nestlings which are vulnerable and susceptible to predators could get risk information both from parents and other birds. Our result showed that cuckoo nestlings distress calls could suppress the begging behavior of three sympatric host nestlings to varying degrees. Our study indicates that host nestlings have the ability to eavesdrop on danger signals expressed by unrelated parasitic nestlings, potentially attributed to innate mechanisms.

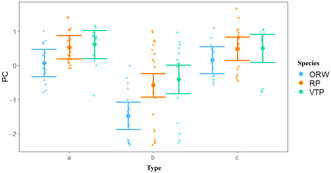

## 1 INTRODUCTION

Breeding is a critical phase in the life cycle of organisms and pivotal for the continuation and expansion of their populations (Bennett & Owens, 2002; Zheng, 2012). For altricial birds, their nestlings are extremely vulnerable during this phase owing to the lack of mobility and defensive behaviors (Martin, 1993, 1995). Predation is a major factor causing breeding failure and offspring loss, as studies suggest that approximately 80% of breeding failures in small birds were attributed to nest predation (Martin, 1993, 1995). Even after leaving the nest, a substantial proportion of young birds experience predation (e.g. Naef‐Daenzer et al., 2001; Zhu et al., 2023). The selective pressure from predation has also considerably influenced the evolutionary relationships in several systems, including the evolution of defense or anti‐predator mechanisms in predator–prey interactions (Caro, 2005).

Adult birds have active anti‐predator strategies. One such example is the oriental reed warbler (*Acrocephalus orientalis*); upon detecting a predator, this species exhibits aggressive behavior and emits alarm calls to attract help from conspecifics and heterospecifics, thereby increasing the chance of expelling the predator from its territory (Wang et al., 2020, 2021). Additionally, they adjust their defense strategies according to their breeding status (Montgomerie & Weatherhead, 1988; Shew et al., 2016; Wang & Yang, 2020). Conversely, nestlings cannot actively flee and are therefore vulnerable to predators. However, nestlings are not entirely unresponsive in the face of danger; they adopt certain behaviors to deter predators. Previous studies have determined that nestlings can obtain information regarding current risks from various sources, including predators (Haff & Magrath, 2010), parents (Jiang, Han, & Yang, 2022; Madden et al., 2005b; Wang et al., 2022b), and other prey species (Jiang, Han, Zhang, et al., 2022), and reduce their activity to hide themselves. For example, nestlings can both recognize different types of alarm calls made by their parents (Magrath et al., 2006; Platzen & Magrath, 2005; Suzuki, 2011) as well as independently assess predator cues, leading to silent responses (Haff & Magrath, 2010). Additionally, when captured by predators or trapped, nestlings emit distress calls (a type of alarm call) (Hörmann et al., 2021; Magrath et al., 2015). Offspring can attract parents with loud distress signals and warn their siblings (Ibáñez‐Álamo et al., 2015; Rohwer et al., 1976). Distress calls share a common characteristic: they typically exhibit low‐frequency noise or high‐intensity broadband signals (Magrath et al., 2015)。.

Wu et al. (2021) discovered that learning, acoustic similarity, and phylogenetic correlations are significant factors influencing bird reactions to distress calls. In certain sympatric species, the acoustic similarity of alarm calls among sympatric species can facilitate a mutual ability to evoke innate responses in birds. For example, white‐browed scrubwren (*Sericornis frontalis*) nestlings responded appropriately to alarm calls from other species that closely resembled their own, but did not respond to dissimilar alarm calls (Haff & Magrath, 2012). Additionally, learning also plays a crucial role in recognizing distress calls. For example, white‐browed scrubwren fledglings learn to respond to alarm calls from different species within a short period after leaving the nest (Haff & Magrath, 2012). Furthermore, phylogenetic relationships themselves may help develop call recognition in different species of animals. For example, anurans that are not sound learners are more effective at recognizing the advertising calls of close relatives than distant relatives (Gingras et al., 2013). Theoretically, it should be beneficial for nestlings to eavesdrop on danger information emitted by species that share common predators, and this behavior may also be common. Studies have found that many adult animals of various species engage in eavesdropping on alarm calls from different species (Yu et al., 2019). However, research on nestlings eavesdropping on danger signals from other prey is limited.

The common cuckoo (hereafter “cuckoo”), a famous obligate brood parasite in the Cuculiformes order, does not build its own nest but instead lays its eggs in the nests of unrelated small passerine birds (hosts). Once the cuckoo eggs are hatched by the host, they will remove the host eggs or chicks out of the nest, thus eliciting the sole care of the adoptive parents (Davies, 2000, 2011; Soler, 2014). In addition, when the adult cuckoo is unable to find a suitable host nest for parasitism (i.e. the host nest is in the late incubation or nestling stage), it will compel the host to construct a new nest by destroying the existing one (farming hypothesis). To date, the cuckoo has been observed to parasitize 276 host species (Mann, 2017) and coexists sympatrically with a diverse range of hosts or potential hosts. Cuckoo nestlings are raised by host foster parents but share no genetic relationship with themand hence their food acquisition and risk transmission come from unrelated hosts (Davies, 2000). The calls of parasitic nestlings are generally louder than those of the host nestlings. The begging calls of a one‐week‐old cuckoo nestling can match the combined volume of a brood of host nestlings to secure sufficient food (Davies et al., 1998). Parasitic nestlings can recognize alarm calls from host adults and respond by suppressing their begging (Davies et al., 2006; Wang et al., 2022a), suggesting that cuckoo nestlings may have integrated into the host's parent‐offspring communication system. Additionally, cuckoo nestlings exhibit extraordinary defensive abilities against predators and emit distress calls (Davies, 2000; Jenner, 1788). Whether long‐term coevolution and coexistence history have enabled host nestlings to recognize these distress calls remains to be determined. Nest parasitism provides an instructive system to explore the recognition of danger signals among nestlings of different species with distant genetic relationships.

The aim of this research was to explore the transmission and recognition of danger information among nestlings of different species with special relationships. To accomplish this aim, in this study, we played back the distress calls of cuckoo nestlings to the nestlings of three sympatric host species (oriental reed warbler, the vinous‐throated parrotbill *Sinosuthora webbiana*, and the reed parrotbill *Paradoxornis heudei*) with varying levels of parasitism pressure by the cuckoo. In addition, we investigated the degree of inhibition of begging behavior in nestlings of each host species. We predicted that the three host species may have evolved an innate ability to recognize the distress calls of cuckoo nestlings because of their long‐term co‐existence in the same habitat, and that this response is independent of phylogenetic relationships.

## 2 MATERIALS AND METHODS

### 2.1 Study site and subjects

The study site is located in the Yongnianwa National Wetland Park, Yongnian District, Hebei Province, China (36°40′–36°41′ N, 114°41′–114°45′ E). This area has a temperate and semi‐humid monsoon continental climate. The Yongnianwa Wetland is a natural depression in the alluvial plain of the Fuyang River; this river is a tributary of the Hai River, which is located at the confluence between the Fuyang and the Zhang rivers. This wetland is only 40.3 m above sea level and is waterlogged year round, making it difficult to access. The average annual rainfall is 527.8 mm, which is primarily concentrated in the summer, and the annual average temperature is 12.9°C. The primary vegetation of this wetland includes reeds *Phragmites australis*, cattails *Typha latifolia*, and other herbaceous plants (Wang & Yang, 2020).

The cuckoo is the most common obligate interspecific brood parasite found in Eurasia (Moksnes et al., 2013; Zheng, 2023). Three sympatric breeding hosts of the cuckoo under different parasitism pressures were selected for this study: the oriental reed warbler, the reed parrotbill, and the vinous‐throated parrotbill. The oriental reed warbler belongs to the family Acrocephalidae within the order Passeriformes. The vinous‐throated parrotbill and the reed parrotbill belong to the family Paradoxornithidae within the same order. All three species of birds can breed in reedbeds (Yang et al., 2015).

The oriental reed warbler is one of the cuckoo's primary hosts (a common host) that has undergone significant coevolution, as evidenced by a high level of co‐adaptation (Li et al., 2016; Yang et al., 2014, 2016, 2017). In this area, the parasitism rate of the oriental reed warbler by the cuckoo is approximately 14.8% (Ma et al., 2018). The reed parrotbill is an incidental host of the cuckoo, with only one case of parasitism observed over 8 years of field research. There has been a long history of co‐evolution between the cuckoo and vinous‐throated parrotbill in Korea (Lee & Yoo, 2004), but no parasitic cases have been found in the local population (personal observation).

### 2.2 Production of playback sounds

In previous experiments, we recorded the distress calls of cuckoo nestlings when their body size was measured. From these recordings, the highest quality audio recordings of cuckoo distress calls from 14 to 16‐day‐old nestlings (cuckoo chicks are easier to record successfully at this age) were selected. In total, three distress call records from three nestlings were used to reduce pseudoreplication. We selected the background noise as the control stimulus (from three selected distress‐call records). We used the Raven Pro software (version 1.4; Cornell Laboratory of Ornithology, Ithaca, NY) to remove low‐frequency noise, edited 30 s segments for playback, saved them in WAV format, and uploaded the files to a Bluetooth player (BV370, SEE ME HERE Electronic Corporation, Shenzhen, China). During the editing of playback sounds, parts overlapping with calls of other birds were removed. The specific method required deleting the section from the beginning of the overlapping phrase to the start of the next phrase to alter the type and rate of the calls as minimally as possible. Several samples of cuckoo distress calls were played back at the same volume, which was approximately 65 dB at 1 m from the speaker.

### 2.3 Procedure of playback experiment

From May to July 2023, we conducted playback experiments on nestlings of three host species aged 6–7 days (oriental reed warbler: *n* = 15; vinous‐throated parrotbill: *n* = 14; reed parrotbill: *n* = 20). The reason for selecting nestlings of this age was that 6–7‐day‐old nestlings exhibit clear begging behavior, respond to human stimuli by begging, and are not excessively fragile (Bernath‐Plaisted & Yasukawa, 2011). To avoid the influence of host parents or other birds, we temporarily placed the 6–7‐day‐old nestlings in collected old nests and brought them to a residence near the research site (less than a 5 min ride by electric bike). The selected host nests contained at least three nestlings each, and one nestling per nest was brought back for the playback experiment (Bernath‐Plaisted & Yasukawa, 2011; Madden et al., 2005b).

The entire procedure of the playback experiment was carried out by Q. Z. The experimental nestlings were placed in the old nests collected after being brought back to the residence. They were allowed to acclimate to the environment and were deprived of food for 40 min before the experiment commenced. The Bluetooth player and recorder (Lotoo L300E, Infomedia Electronic Technology Corporation, Beijing, China) were positioned within a distance of 0.5 m from the nest, and the digital video recorder was placed 1 m from the nest to document the entire experiment. Each nestling was tested individually. Prior each playback, we gently touched the nest with our hands to prompt the nestlings to produce normal begging calls, and repeated this gentle touching every 3 s. Simultaneously, the recorder and digital video recorder were utilized for a recording duration of 30 s to compare their behaviors during playback with their natural behaviors. After allowing the nestling to calm for 1 min, two 30 s sound clips were played back to each nestling: the cuckoo's distress calls (73.00 ± 16.52 calls/min), and background noise, in a random sequence. There was a 5 min interval between the playback of the two sounds (Madden et al., 2005a, 2005b). During the playback, the nest was also touched gently every 3 s to maintain consistency with the touch above that induced begging, and the begging behavior of the nestlings was recorded. After the playback was completed, the nestlings were promptly returned to the original nest, which was numbered to avoid repeated playback to the same nestlings. Upon revisiting the nest the following day, we observed the parents near the nest and all the chicks appeared to be in good condition. We did not choose to play back the distress calls of the host nestlings here because Jiang, Han, Zhang, et al. (2022) already used this methodology in previous studies, so we instead compared our results with his (see Section 4).

The quantification of the begging behavior of the nestlings was conducted indoors by T. Z., following which J. W. reviewed the quantification results. If any questionable data was identified, the video was reviewed again to ensure accuracy and consistency in the analysis. The recorded behaviors of the nestlings included the following: total begging time, total number of calls, begging score (0 = no begging, 1 = opening mouth, bent tarsus, 2 = includes 1 plus stretched neck, 3 = includes 2 plus extended tarsus, 4 = includes 3 plus wing flapping or shaking), number of bouts of begging (total number of begging bouts in 30 s), and crouched behavior (yes/no).

### 2.4 Call analyses

We also selected the distress calls of the host nestlings (oriental reed warbler: *n* = 13; vinous‐throated parrotbill: *n* = 8; reed parrotbill: *n* = 8) at 8–9 days of age with a higher recording quality from the previous recordings. We quantified the lowest frequency, highest frequency, bandwidth, peak frequency, and duration of distress calls in nestlings from three host species and cuckoo nestlings (*n* = 12) to investigate the acoustic similarity between them.

### 2.5 Statistical analyses

Data analysis was performed using IBM SPSS 26.0 for Windows (International Business Machines Corporation, New York, USA). Initially, a principal component analysis (PCA) was performed on the host nestlings' total begging time, number of bouts of begging, total number of calls, begging score, and the crouched behavior (yes/no). One principal component (PC1) with an eigenvalue >1 was extracted from nestlings, and we used this factor to represent the nestling begging behavior. The generalized linear mixed model (GLMM) was used to estimate the effects of distress calls of cuckoo on the PC1 of begging behavior in the nestlings of the three species; nest identity and nestling weight were treated as random effects. The type (nestlings' natural begging, cuckoo nestling distress calls, and background noise), playback order, nestling species (oriental reed warbler, vinous‐throated parrotbill, and reed parrotbill), interaction between type and playback order, and interaction between type and nestling species were tested as fixed factors in the GLMM. Pairwise comparisons were conducted using the least significant difference method (LSD).

We further calculated the degree of inhibition of the nestlings' begging behavior; more specifically, the difference between the begging behavior before and after playback of the distress call divided by the begging behavior before playback. In this analysis, we selected three original variables of the begging behavior of the nestlings: total begging time, begging score, and total number of calls. We next evaluated the inhibition level for each species after exposure to the distress calls of the cuckoo nestlings. There are several reasons for choosing the three original variables: First, utilizing the begging behavior PC1 to assess the degree of inhibition would result in a loss of the underlying original information. Second, the crouched behavior (yes/no) is a categorical variable, and the number of begging bouts has a limited capacity to capture the complexity of begging behaviors. Because the data of each group did not adhere to the normal distribution, we employed a non‐parametric test to investigate whether there were significant differences in the degree of inhibition of nestlings among the three host species. Specifically, we conducted multiple comparisons using the Kruskal–Wallis test.

Finally, a discriminant analysis was utilized to examine the acoustic parameters of distress calls produced by cuckoo nestlings and nestlings of three host species and thus investigate if acoustic similarity influenced the response to distress calls from cuckoo nestlings. All statistical tests were two‐tailed, with *p* = .05 as the level of significance. Unless specifically stated, data are presented as mean ± SD.

## 3 RESULTS

**FIGURE 1:**
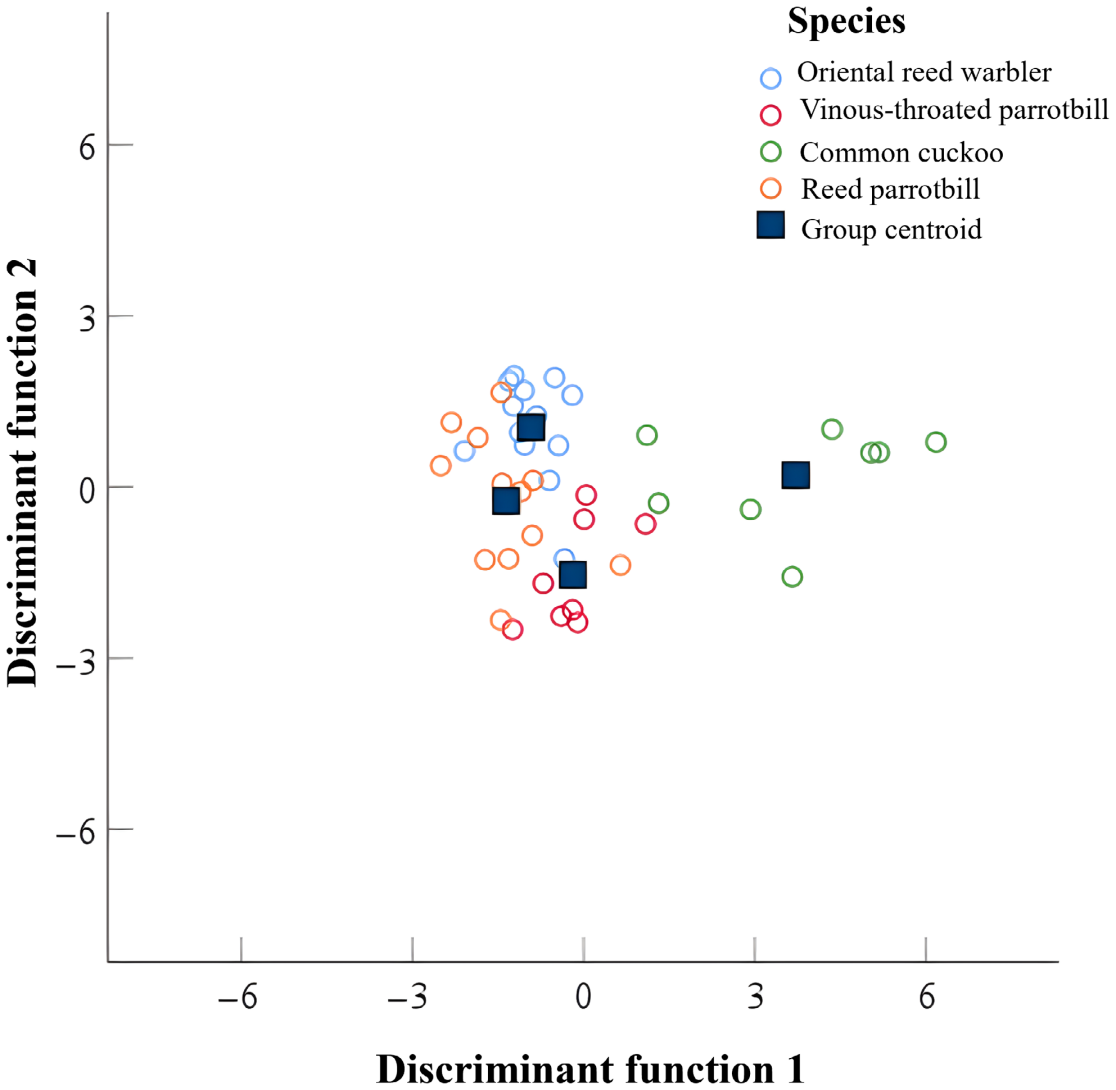
Typical diagrams of distress calls of four species of nestlings separated from discriminant function analysis.

The discriminant analysis revealed that the probability of accurately classifying the distress calls of the four species of nestlings based on the five acoustic parameters was 75.6%.The distress calls of cuckoo nestlings possessed have distinct acoustic properties that did not overlap with the distress calls of the three host species' nestlings (Figure 1).

Playback experiments were conducted on 15 oriental reed warbler nestlings, 14 vinous‐throated parrotbill nestlings, and 20 reed parrotbill nestlings. The PCA was conducted on the host nestlings' total begging time, number of bouts of begging, total number of calls, begging score, and the crouched behavior (yes/no) (KMO = 0.759, *p* < .001). The result extracted one principal component with an eigenvalue >1.0, explaining 61.68% of the total variance in the response data. Factor loadings for the principal components are listed in Table 1.

**TABLE 1:** Components extracted by principal component analysis for behavioral responses in nestlings.

Behavioral response	PC
Total begging time	0.898
Begging score	0.838
Crouched behavior (yes/no)	−0.814
Total number of calls	0.788
Number of bouts of begging	0.540

**FIGURE 2:**
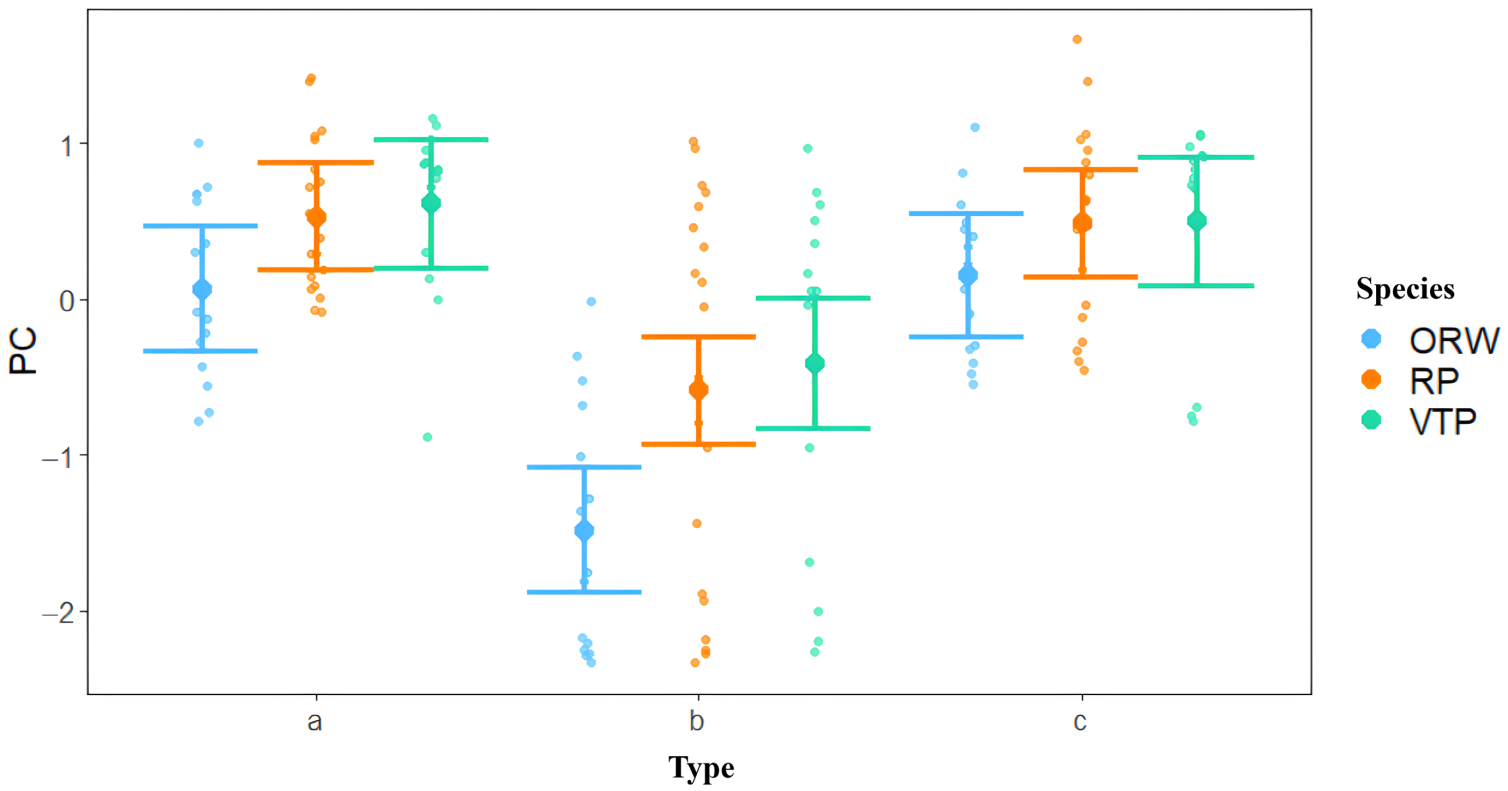
Begging responses of nestlings of three sympatric host species based on principle component analysis (ORW, oriental reed warbler; RP, reed parrotbill; VTP, vinous‐throated parrotbill) to different playback sounds. (a) Natural begging; (b) common cuckoo nestling distress calls, and (c) background noise.

The GLMM results indicated that the two variables of type and species had significant effects on the behavioral responses of nestlings (*F*1_1,133_ = 68.095, *p*1 < .001; *F*2_2,133_ = 6.911, *p*2 = .001; Table 2). Multiple comparisons showed that playback of the cuckoo nestling distress calls significantly suppressed the begging behaviors of the three host species. Compared with natural begging of the host nestlings and the playback of background noise, playback of the distress calls resulted in a significantly lower begging behavior in host nestlings (*t*1 = 8.531, *p*1 < .001; *t*2 = 8.252, *p*2 < .001). However, no significant difference was observed in the begging behavior of host nestlings in their natural begging and response to playback of background noise (*t* = −0.262, *p* = .794) (Figure 2). Among the species, the begging behavior of the oriental reed warbler was significantly lower than that of vinous‐throated parrotbill and reed parrotbill (*t*1 = −3.609, *p*1 < .001; *t*2 = −2.678, *p*2 = .008). No significant difference was observed in the begging behavior PC between vinous‐throated parrotbill and reed parrotbill, although the former had a higher begging behavior value than the latter (*t* = 0.909, *p* = .365) (Figure 2).

**TABLE 2:** Generalized linear mixed model for behavioral responses in nestlings.

	*F*	df1	df2	*p*
Type	68.095	1	133	**<.001**
Species	6.911	2	133	**.001**
Order	0.547	1	173	.461
Type × Order	1.013	1	133	.316
Type × Species	1.720	4	133	.316

*Note*: Significant effects are in bold.

Additionally, in using the original variable of begging behavior to measure the degree of inhibition, we discovered a notable difference in the degree of inhibition in the total number of calls among the three host species after they were exposed to the distress calls of cuckoo nestlings (oriental reed warbler: 0.909 ± 0.241, vinous‐throated parrotbill: 0.323 ± 0.943, reed parrotbill: 0.751 ± 0.372; *p* = .019). The results of the multiple comparisons showed that the degree of inhibition in the begging behavior of oriental reed warbler nestlings was significantly higher than that of the vinous‐throated parrotbill nestlings (*p* = .016). There were no significant differences among other groups (All *p* > .05). However, after hearing the distress calls of cuckoo nestlings, there was no significant difference in the total begging time and begging score among the three host species nestlings (begging score: *p* = .311; total begging time: *p* = .113).

## 4 DISCUSSION

Our results indicate that the distress calls of cuckoo nestlings significantly suppressed the begging behaviors of hosts, suggesting that host nestlings used the distress call of cuckoo nestlings as a danger signal, although the degree of suppression varied. We found that the main host of the cuckoo, the oriental reed warbler, had the strongest response to the distress call and the strongest inhibition in the number of calls. Our results suggest that the host nestlings' response to the distress calls of cuckoo nestlings may be related to their innate mechanisms.

Nest predation is one of the most significant selective pressures in nature (Martin, 1995). Compared with adult birds, altricial nestlings in the nest lack mobility yet are not entirely defenseless against predation risk and have evolved various survival strategies (e.g. Canestrari et al., 2014; Wang et al., 2022a). Distress calls of nearby heterospecific nestlings often represent an immediate and closer predation risk in the environment, and eavesdropping on and recognizing these signals from heterospecific nestlings are beneficial. Jiang, Han, Zhang, et al. (2022) found that two sympatric nestling species suppressed their begging behavior in response to the distress calls of heterospecific nestlings, similar to their reactions to adult alarm calls (Jiang, Han, & Yang, 2022; Wang et al., 2022a). Similarly, in this study, the three sympatric hosts of the cuckoo specifically responded to the cuckoo's distress calls by significantly suppressing and reducing their begging, which was consistent with our prediction; this indicates that host nestlings can eavesdrop on the danger information represented by cuckoo distress calls. In the study area, where predation rates are high and several host nests intermingled in the local reed wetlands (Ma et al., 2021), parents are not always present at the nest during predation events. Therefore, obtaining predation risk information solely from adult alarm calls is insufficient and eavesdropping on distress calls of sympatric heterospecific nestlings is beneficial.

Previous research has shown that innate mechanisms, learning, acoustic similarity, and phylogenetic relatedness strongly influence responses to distress calls (Haff & Magrath, 2012, 2013; Huang et al., 2018; Wu et al., 2021). In the present study, because the cuckoo and the three host species were not closely related, host nestlings' responses to distress calls were not driven by phylogenetic factors. According to the results of our discriminant analysis, there was no similarity found between the acoustic parameters of the distress calls of cuckoo nestlings and those of the three host species, suggesting that acoustic similarity may not have influenced the response to cuckoo distress calls either. Jiang, Han, Zhang, et al. (2022) reported similar findings. Unlike the findings of the present study, at a younger age (5–6 days), white‐browed scrubwren nestlings respond only to heterospecific alarm calls that are acoustically similar to the conspecifics (Haff & Magrath, 2012). The study area was subjected to greater predation pressure, which may have resulted in an innate response of host nestlings toward the distress calls of cuckoo nestlings. The distress calls of nestlings are typically characterized by harsh, abrupt, and broadband sounds. The nonlinear features of certain distress signals could constitute the reason why nestlings instinctively react without the need for learning (Rendall et al., 2009; Seiler et al., 2013). Similarly, Biedenweg et al. (2011) discovered that western gray kangaroos (*Macropus fuliginosus*) react more strongly to synthetic sounds with nonlinearities than to those without, suggesting that nonlinear properties may be represent an important component of bird responses.

The findings of this study, along with those of Jiang, Han, Zhang, et al. (2022), demonstrate that host nestlings can eavesdrop on both the distress calls of local host nestlings and cuckoo nestlings. However, differing from the present this study, Jiang, Han, Zhang, et al. (2022) found that both nestlings reduced their begging in response to the distress calls, but the extent to which the two host nestlings suppressed their begging behavior was unknown. Our study revealed that oriental reed warbler nestlings exhibited a significantly greater suppression of their total number of calls after being exposed to the distress calls of cuckoo nestlings compared to vinous‐throated parrotbill nestlings. We propose several potential reasons for this observed difference. First, it may be related to the double pressure of predation and parasitism on the oriental reed warbler. Although the parasite does not directly harm the adult bird, if it fails to locate a suitable host nest, it may resort to destroying the host nest during late incubation or when nestlings are present. This compels the host to construct a new nest, ultimately raising the likelihood of future infestations as proposed by the farming hypothesis (Soler et al., 2017). Furthermore, other studies have provided evidence that adult cuckoos kill their host nestlings (Šulc et al., 2020). Therefore, in addition to the predation pressure, the oriental reed warbler nestlings also suffer from the additional stress caused by cuckoo farming behavior. Moreover, the oriental reed warbler boasts the largest population in the study area, making it more susceptible to predation pressure compared to the other two species (Ma et al., 2021). Furthermore, due to its larger size, the oriental reed warbler may be more easily detected by predators compared to the vinous‐throated parrotbill. Previous studies have shown that replaying the begging calls of nestlings attracts the attention of predators (Dearborn, 1999; but see Yasukawa, 2016), so reducing the number of begging calls after hearing distress calls is beneficial for the nestlings' own survival.

In conclusion, our study showed that nestlings of three sympatric host species can eavesdrop on the danger information communicated by parasitic nestlings (cuckoos) and exhibit suppressed begging behavior, which may be related to innate mechanisms. In addition, oriental reed warbler nestlings had the highest degree of inhibition in the number of calls after being exposed to the distress calls of cuckoo nestlings, which may be linked to the combined pressures of predation and parasitism.

## AUTHOR CONTRIBUTIONS

**Jiaojiao Wang:** Conceptualization (lead); data curation (equal); funding acquisition (equal); writing – original draft (equal). **Qindong Zhou:** Formal analysis (equal); investigation (lead). **Taijun Zuo:** Data curation (equal); formal analysis (equal). **Longwu Wang:** Funding acquisition (equal); writing – review and editing (equal). **Laikun Ma:** Formal analysis (equal); funding acquisition (equal); writing – original draft (equal). **Jianhua Hou:** Writing – review and editing (equal).

## FUNDING INFORMATION

This work was funded by the National Natural Science Foundation of China (32301295 to JW, 32101242 to LM and 32260253 to LW) and High‐Level Talents Research Start‐Up Project of Hebei University (521100222044 to JW).

## CONFLICT OF INTEREST STATEMENT

The authors declare that they have no competing interests.

## Supporting information


Audios S1–S3.

## Data Availability

The data related to this study has been submitted simultaneously as supporting material and is also available from the corresponding author upon reasonable request.

## References

[ece311437-bib-0001] Bennett, P. M. , & Owens, I. P. (2002). Evolutionary ecology of birds: Life histories, mating systems and extinction. Oxford University Press.

[ece311437-bib-0002] Bernath‐Plaisted, J. , & Yasukawa, K. (2011). Effect of alarm calling by male red‐winged blackbirds on nestling begging and female provisioning behavior. Journal of Field Ornithology, 82, 395–405.

[ece311437-bib-0003] Biedenweg, T. A. , Parsons, M. H. , Fleming, P. A. , & Blumstein, D. T. (2011). Sounds scary? Lack of habituation following the presentation of novel sounds. PLoS One, 6, e14549. 21267451 10.1371/journal.pone.0014549 PMC3022648

[ece311437-bib-0004] Canestrari, D. , Bolopo, D. , Turlings, T. C. J. , Rőder, G. , Marcos, J. M. , & Baglione, V. (2014). From parasitism to mutualism: Unexpected interactions between a cuckoo and its host. Science, 343, 1350–1352. 24653032 10.1126/science.1249008

[ece311437-bib-0005] Caro, T. M. (2005). Antipredator defenses in birds and mammals. University of Chicago Press.

[ece311437-bib-0006] Davies, N. B. , Kilner, R. M. , & Noble, D. G. (1998). Nestling cuckoos, *Cuculus canorus*, exploit hosts with begging calls that mimic a brood. Proceedings of the Royal Society of London, Series B: Biological Sciences, 265, 673–678.

[ece311437-bib-0007] Davies, N. B. (2000). Cuckoos, cowbirds, and other cheats. T. & AD Poyser.

[ece311437-bib-0008] Davies, N. B. , Madden, J. R. , Butchart, S. H. M. , & Rutila, J. (2006). A host‐race of the cuckoo *Cuculus canorus* with nestlings attuned to the parental alarm calls of the host species. Proceedings of the Royal Society B: Biological Sciences, 273, 693–699. 10.1098/rspb.2005.3324 PMC1560078 16608688

[ece311437-bib-0009] Davies, N. B. (2011). Cuckoo adaptations: Trickery and tuning. Journal of Zoology, 284, 1–14.

[ece311437-bib-0010] Dearborn, D. C. (1999). Brown‐headed cowbird nestling vocalizations and risk of nest predation. Auk, 116, 448–457.

[ece311437-bib-0011] Gingras, B. , Mohandesan, E. , Boko, D. , & Fitch, W. T. (2013). Phylogenetic signal in the acoustic parameters of the advertisement calls of four clades of anurans. BMC Evolutionary Biology, 13, 134. 23815403 10.1186/1471-2148-13-134 PMC3703296

[ece311437-bib-0012] Haff, T. M. , & Magrath, R. D. (2010). Vulnerable but not helpless: Nestlings are fine‐tuned to cues of approaching danger. Animal Behaviour, 79, 487–496.

[ece311437-bib-0013] Haff, T. M. , & Magrath, R. D. (2012). Learning to listen? Nestling response to heterospecific alarm calls. Animal Behaviour, 84, 1401–1410.

[ece311437-bib-0014] Haff, T. M. , & Magrath, R. D. (2013). Eavesdropping on the neighbours: Fledglings learn to respond to heterospecific alarm calls. Animal Behaviour, 85, 411–418.

[ece311437-bib-0015] Hörmann, D. , Tschapka, M. , Rose, A. , & Knörnschild, M. (2021). Distress calls of nectarivorous bats (*Glossophaga soricina*) encode individual and species identity. Bioacoustics, 30, 253–271.

[ece311437-bib-0016] Huang, X. , Metzner, W. , Zhang, K. , Wang, Y. , Luo, B. , Sun, C. , Jiang, T. , & Feng, J. (2018). Acoustic similarity elicits responses to heterospecific distress calls in bats (Mammalia: Chiroptera). Animal Behaviour, 146, 143–154.

[ece311437-bib-0017] Ibáñez‐Álamo, J. D. , Magrath, R. D. , Oteyza, J. C. , Chalfoun, A. D. , Haff, T. M. , Schmidt, K. A. , Thomson, R. L. , & Martin, T. E. (2015). Nest predation research: Recent findings and future perspectives. Journal of Ornithology, 156, 247–262.

[ece311437-bib-0018] Jenner, E. (1788). Observations on the natural history of the cuckoo. Philosophical Transactions of the Royal Society of London. Series B, Biological Sciences, 78, 219–237.

[ece311437-bib-0019] Jiang, Y. , Han, J. , & Yang, C. (2022). Anti‐predation responses to conspecific versus heterospecific alarm calls by the nestlings of two sympatric birds. Animals, 12, 2156. 36009746 10.3390/ani12162156 PMC9404724

[ece311437-bib-0020] Jiang, Y. , Han, J. , Zhang, Z. , Chen, X. , & Yang, C. (2022). Parent‐offspring and inter‐offspring responses to conspecific versus heterospecific distress calls in 2 sympatric birds. Current Zoology, 68, 700–707. 36743226 10.1093/cz/zoab103 PMC9892787

[ece311437-bib-0021] Lee, J. W. , & Yoo, J. C. (2004). Effect of host egg color dimorphism on interactions between the vinous‐throated parrotbill (*Paradoxornis webbianus*) and common cuckoo (*Cuculus canorus*). Korean Journal of Biological Sciences, 8, 77–80.

[ece311437-bib-0022] Li, D. , Zhang, Z. , Grim, T. , Liang, W. , & Stokke, B. G. (2016). Explaining variation in brood parasitism rates between potential host species with similar habitat requirements. Evolutionary Ecology, 30, 905–923.

[ece311437-bib-0023] Ma, L. , Yang, C. , Liu, J. , Zhang, J. , Liang, W. , & Møller, A. P. (2018). Costs of breeding far away from neighbors: Isolated host nests are more vulnerable to cuckoo parasitism. Behavioural Processes, 157, 327–332. 30059764 10.1016/j.beproc.2018.07.017

[ece311437-bib-0024] Ma, L. , Yang, C. , & Liang, W. (2021). Nest‐site choice and breeding success among four sympatric species of passerine birds in a reedbed‐dominated wetland. Journal of Resources and Ecology, 12, 22–29.

[ece311437-bib-0025] Madden, J. R. , Kilner, R. M. , & Davies, N. B. (2005a). Nestling responses to adult food and alarm calls: 2. Cowbirds and red‐winged blackbirds reared by eastern phoebe hosts. Animal Behaviour, 70, 629–637.

[ece311437-bib-0026] Madden, J. R. , Kilner, R. M. , & Davies, N. B. (2005b). Nestling responses to adult food and alarm calls: 1. Species‐specific responses in two cowbird hosts. Animal Behaviour, 70, 619–627.

[ece311437-bib-0027] Magrath, R. D. , Platzen, D. , & Kondo, J. (2006). From nestling calls to fledgling silence: Adaptive timing of change in response to aerial alarm calls. Proceedings of the Royal Society B: Biological Sciences, 273, 2335–2341. 10.1098/rspb.2006.3610 PMC1636086 16928636

[ece311437-bib-0028] Magrath, R. D. , Haff, T. M. , Fallow, P. M. , & Radford, A. N. (2015). Eavesdropping on heterospecific alarm calls: From mechanisms to consequences. Biological Reviews of the Cambridge Philosophical Society, 90, 560–586. 24917385 10.1111/brv.12122

[ece311437-bib-0029] Mann, C. F. (2017). A taxonomic review of obligate and facultative interspecific avian brood parasitism. In M. Soler (Ed.), Avian brood parasitism (pp. 61–92). Springer.

[ece311437-bib-0030] Martin, T. E. (1993). Nest predation and nest sites. Bioscience, 43, 523–532.

[ece311437-bib-0031] Martin, T. E. (1995). Avian life history evolution in relation to nest sites, nest predation, and food. Ecological Monographs, 65, 101–127.

[ece311437-bib-0032] Moksnes, A. , FossØY, F. , RØSkaft, E. , & Stokke, B. G. (2013). Reviewing 30 years of studies on the common cuckoo: Accumulated knowledge and future perspectives. Chinese Birds, 4, 3–14.

[ece311437-bib-0033] Montgomerie, R. D. , & Weatherhead, P. J. (1988). Risk and rewards of nest defence by parent birds. The Quarterly Review of Biology, 63, 167–187.

[ece311437-bib-0034] Naef‐Daenzer, B. , Widmer, F. , & Nuber, M. (2001). Differential post‐fledging survival of great and coal tits in relation to their condition and fledging date. Journal of Animal Ecology, 70, 730–738.

[ece311437-bib-0035] Platzen, D. , & Magrath, R. D. (2005). Adaptive differences in response to two types of parental alarm call in altricial nestlings. Proceedings of the Royal Society B: Biological Sciences, 272, 1101–1106. 10.1098/rspb.2005.3055 PMC1559813 16024370

[ece311437-bib-0036] Rendall, D. , Owren, M. J. , & Ryan, M. J. (2009). What do animal signals mean? Animal Behaviour, 78, 233–240.

[ece311437-bib-0037] Rohwer, S. , Fretwell, S. D. , & Tuckfield, R. C. (1976). Distress screams as a measure of kinship in birds. American Midland Naturalist, 96, 418–430.

[ece311437-bib-0038] Seiler, M. , Schwitzer, C. , Gamba, M. , & Holderied, M. W. (2013). Interspecific semantic alarm call recognition in the solitary Sahamalaza sportive lemur, *Lepilemur sahamalazensis* . PLoS One, 8, e67397. 23825658 10.1371/journal.pone.0067397 PMC3692490

[ece311437-bib-0039] Shew, J. J. , van der Merwe, J. , Schauber, E. M. , Tallitsch, B. K. , & Nielsen, C. K. (2016). A classic question revisited in red‐winged blackbirds: Disentangling confounding hypotheses surrounding parental investment theory and nest defense intensity. Behavioral Ecology and Sociobiology, 70, 1843–1856.

[ece311437-bib-0040] Soler, M. (2014). Long‐term coevolution between avian brood parasites and their hosts. Biological Reviews of the Cambridge Philosophical Society, 89, 688–704. 24330159 10.1111/brv.12075

[ece311437-bib-0041] Soler, M. , Pérez‐Contreras, T. , & Soler, J. J. (2017). Brood parasites as predators: Farming and mafia strategies. In M. Soler (Ed.), Avian brood parasitism (pp. 271–286). Springer: Berlin/Heidelberg.

[ece311437-bib-0042] Šulc, M. , Štětková, G. , Jelínek, V. , Czyż, B. , Dyrcz, A. , Karpińska, O. , Kamionka‐Kanclerska, K. , Rowiński, P. , Maziarz, M. , Gruszczyński, A. , Hughes, A. E. , & Honza, M. (2020). Killing behaviour of adult brood parasites. Behaviour, 157, 1099–1111.

[ece311437-bib-0043] Suzuki, T. N. (2011). Parental alarm calls warn nestlings about different predatory threats. Current Biology, 21, R15–R16. 21215927 10.1016/j.cub.2010.11.027

[ece311437-bib-0044] Wang, J. , Ma, L. , Liang, W. , & Yang, C. (2020). Responses of cuckoo hosts to alarm signals of different nest intruders in non‐nesting areas. Zoological Research, 41, 345–350. 32212428 10.24272/j.issn.2095-8137.2020.030 PMC7231467

[ece311437-bib-0045] Wang, J. , & Yang, C. (2020). Specific responses of cuckoo hosts to different alarm signals according to breeding stage: A test of the offspring value hypothesis. Current Zoology, 66, 649–655. 33391364 10.1093/cz/zoaa021 PMC7769587

[ece311437-bib-0046] Wang, J. , Ma, L. , Chen, X. , & Yang, C. (2021). Behavioral and acoustic responses of the oriental reed warbler (*Acrocephalus orientalis*), at egg and nestling stages, to the common cuckoo (*Cuculus canorus*). Frontiers in Ecology and Evolution, 9, 705748.

[ece311437-bib-0047] Wang, J. , Ma, L. , Chen, X. , & Yang, C. (2022a). Common cuckoo nestling adapts its begging behavior to the alarm signaling system of a host. Frontiers in Ecology and Evolution, 10, 830441.

[ece311437-bib-0048] Wang, J. , Ma, L. , Chen, X. , & Yang, C. (2022b). Female cuckoo calls deceive their hosts by evoking nest‐leaving behavior: Variation under different levels of parasitism. Animals, 12, 1990. 35953979 10.3390/ani12151990 PMC9367515

[ece311437-bib-0049] Wu, Y. , Petrosky, A. L. , Hazzi, N. A. , Woodward, R. L. , & Sandoval, L. (2021). The role of learning, acoustic similarity and phylogenetic relatedness in the recognition of distress calls in birds. Animal Behaviour, 175, 111–121.

[ece311437-bib-0050] Yang, C. , Li, D. , Wang, L. , Liang, G. , Zhang, Z. , & Liang, W. (2014). Geographic variation in parasitism rates of two sympatric cuckoo hosts in China. Zoological Research, 35, 67–71. 24470456 10.11813/j.issn.0254-5853.2014.1.067 PMC5042954

[ece311437-bib-0051] Yang, C. , Wang, L. , Cheng, S.‐J. , Hsu, Y.‐C. , Stokke, B. G. , Roskaft, E. , Moksnes, A. , Liang, W. , & Moller, A. P. (2015). Deficiency in egg rejection in a host species as a response to the absence of brood parasitism. Behavioral Ecology, 26, 406–415.

[ece311437-bib-0052] Yang, C. , Wang, L. , Liang, W. , & Møller, A. P. (2016). Egg recognition as antiparasitism defence in hosts does not select for laying of matching eggs in parasitic cuckoos. Animal Behaviour, 122, 177–181.

[ece311437-bib-0053] Yang, C. , Wang, L. , Liang, W. , & Møller, A. P. (2017). How cuckoos find and choose host nests for parasitism. Behavioral Ecology, 28, 859–865.

[ece311437-bib-0054] Yasukawa, K. (2016). Do begging calls from nestling red‐winged blackbirds (*Agelaius phoeniceus*) increase nest predation? The Wilson Journal of Ornithology, 128, 879–884.

[ece311437-bib-0055] Yu, J. , Lu, H. , Sun, W. , Liang, W. , Wang, H. , & Møller, A. P. (2019). Heterospecific alarm‐call recognition in two warbler hosts of common cuckoos. Animal Cognition, 22, 1149–1157. 31506795 10.1007/s10071-019-01307-9 PMC6834739

[ece311437-bib-0056] Zheng, G. (2012). Ornithology (2nd ed.). Beijing Normal University Press.

[ece311437-bib-0057] Zheng, G. (2023). A checklist on the classification and distribution of the birds of China (4rd ed.). Science Press.

[ece311437-bib-0058] Zhu, G. , Zheng, M. , Lyu, S. , & Ma, L. (2023). Report of a magpie preying on a post‐fledgling Daurian redstart. Ecology and Evolution, 13, e10412. 37565028 10.1002/ece3.10412 PMC10410626

